# *NQO1* polymorphism and susceptibility to ischemic stroke in a Chinese population

**DOI:** 10.1186/s12920-024-01992-7

**Published:** 2024-08-22

**Authors:** Min Wang, Ying Shen, Yuan Gao, Huaqiu Chen, Fuhui Duan, Siying Li, Guangming Wang

**Affiliations:** 1 School of Clinical Medicine, Dali University, Dali, Yunnan 671000 PR China; 2 The First Hospital of Liangshan, Xichang, Sichuan 615000 PR China; 3 School of Clinical Medicine, Zhengzhou University, Zhengzhou, Henan 450001 PR China; 4 Xichang People’s Hospital, Xichang, Sichuan 615000 PR China; 5 Center of Genetic Testing, The First Affiliated Hospital of Dali University, Dali, Yunnan 671000 PR China

**Keywords:** *NQO1*, Ischemic stroke, Genetic polymorphism, Genetic susceptibility, Enzyme-linked immunosorbent assay

## Abstract

**Background:**

Ischemic stroke (IS) is a major cause of death and disability worldwide. Genetic factors are important risk factors for the development of IS. The quinone oxidoreductase 1 gene (*NQO1*) has antioxidant, anti-inflammatory, and cytoprotective properties. Thus, in this study, we investigated the relationship between NQO1 gene polymorphism and the risk of IS.

**Methods:**

Peripheral blood was collected from 143 patients with IS and 124 the control groups in Yunnan, China, and *NQO1* rs2917673, rs689455, and rs1800566 were genotyped. Logistic regression was used to analyze the relationship between the three *NQO1* loci and IS susceptibility. The difference in the expression levels of *NQO1* between the control groups and IS groups was verified using public databases and enzyme-linked immunosorbent assay.

**Results:**

The rs2917673 locus increased the risk of IS by 2.375 times in TT genotype carriers under the co-dominance model compared with CC carriers and was statistically associated with the risk of IS (OR = 2.375, 95% CI = 1.017–5.546, *P* = 0.046). In the recessive model, TT genotype carriers increased IS risk by 2.407 times compared with CC/CT carriers and were statistically associated with the risk of IS (OR = 2.407, 95% CI = 1.073–5.396, *P* = 0.033).

**Conclusions:**

*NQO1* rs2917673 polymorphism is significantly associated with IS. Mutant TT carriers are risk factors for IS.

**Supplementary Information:**

The online version contains supplementary material available at 10.1186/s12920-024-01992-7.

## Background

Stroke is divided into ischemic stroke (IS) and hemorrhagic stroke. In most cases, stroke is caused by a sudden blockage of arteries and can damage neurological function [1]. Acute IS often occurs suddenly and progresses rapidly, and intravenous thrombolysis using a recombinant tissue plasminogen activator is the most common treatment. However, the treatment window is only 3 h, and stroke is the second-leading cause of death [2]. IS after cerebral artery occlusion is a leading cause of chronic disability worldwide [3]. The 2020 Estimated Burden of Stroke in China Study shows that IS accounts for 15.5% of all stroke events [4]. Furthermore, the incidence of stroke among young people is increasing. The high incidence and large number of patients place a huge burden on medical resources and society [5, 6]. Stroke is a multifactorial, complex neurological disease involving clinical, environmental, and genetic factors [7]. The common risk factors for stroke include age, gender, smoking, alcohol abuse, diabetes, hyperlipidemia, and hyperhomocysteinemia [8, 9]. In addition to common risk factors, the International Stroke Genetics Consortium states that genetic factors may account for 50% of an individual’s risk of stroke and may play a key regulatory role in pathophysiological processes, such as brain cell necrosis and ischemia–reperfusion injury [10–13]. Therefore, there is an urgent need to further explore the relationship between genetic polymorphisms and IS [14].

The human *NQO1* gene, also known as the DT-flavosome, comprises six exons separated by five introns. It is located on chromosome 16 and is present in human endothelial tissue. The gene encodes a flavoprotein called NAD(P)H dehydrogenase quinone 1 [15–17]. NQO1 is an obligate two-electron reductase characterized by its ability to utilize NADH or NADPH as a reduction cofactor and its inhibitory effect as dicoumarol; it reacts with NADH to catalyze the two-electron reduction of quinones into redox-stable hydroquinone, thereby increasing intracellular NAD + levels, which in turn prevents the formation of free radicals [18]. NQO1 exerts antioxidant, anti-inflammatory, and cytoprotective effects on the nervous system [19–24]. Studies have reported that individuals carrying two mutant genomic alleles have no NQO1 activity, whereas heterozygous individuals with one mutant allele have low to moderate NQO1 activity compared with wild-type individuals [15]. Multiple studies have shown that *NQO1* polymorphisms are associated with the risk of cardiovascular diseases [16]. Polymorphic forms of *NQO1* (p.P187S) are associated with an increased cancer risk and certain neurological diseases, possibly because of their role in antioxidant defense. NQO1 serves as a good model for studying loss-of-function mechanisms in genetic diseases and improving strategies to distinguish neutral from pathogenic variants in whole-genome sequencing studies [19]. Genetic factors have been identified as contributing to stroke susceptibility through high-throughput genotyping technology [25]. To date, the relationship between *NQO1* polymorphisms and susceptibility to IS has not been reported. Therefore, it is important to study the relationship between *NQO1* polymorphisms and the risk of IS.

SNP genotyping technologies include TaqManTM hybridization probes and SNaPshot micro-sequencing methods [26]. Among them, SNaPshot based on micro-sequencing technology is superior to other technologies because of its high sensitivity, strong multiplexing ability, and no need for additional equipment [27].

We explored the relationship between three *NQO1* polymorphisms—rs2917673, rs689455, and rs1800566—and the risk of IS in the Yunnan population of China. Logistic regression analysis using odds ratios (OR) and 95% confidence intervals (CI) was used to evaluate the relationship between *NQO1* polymorphisms and IS susceptibility. The present study is expected to provide valuable theoretical data to support the early prevention, diagnosis, and treatment of IS.

## Materials and methods

### Study population and sample collection

**Fig. 1:**
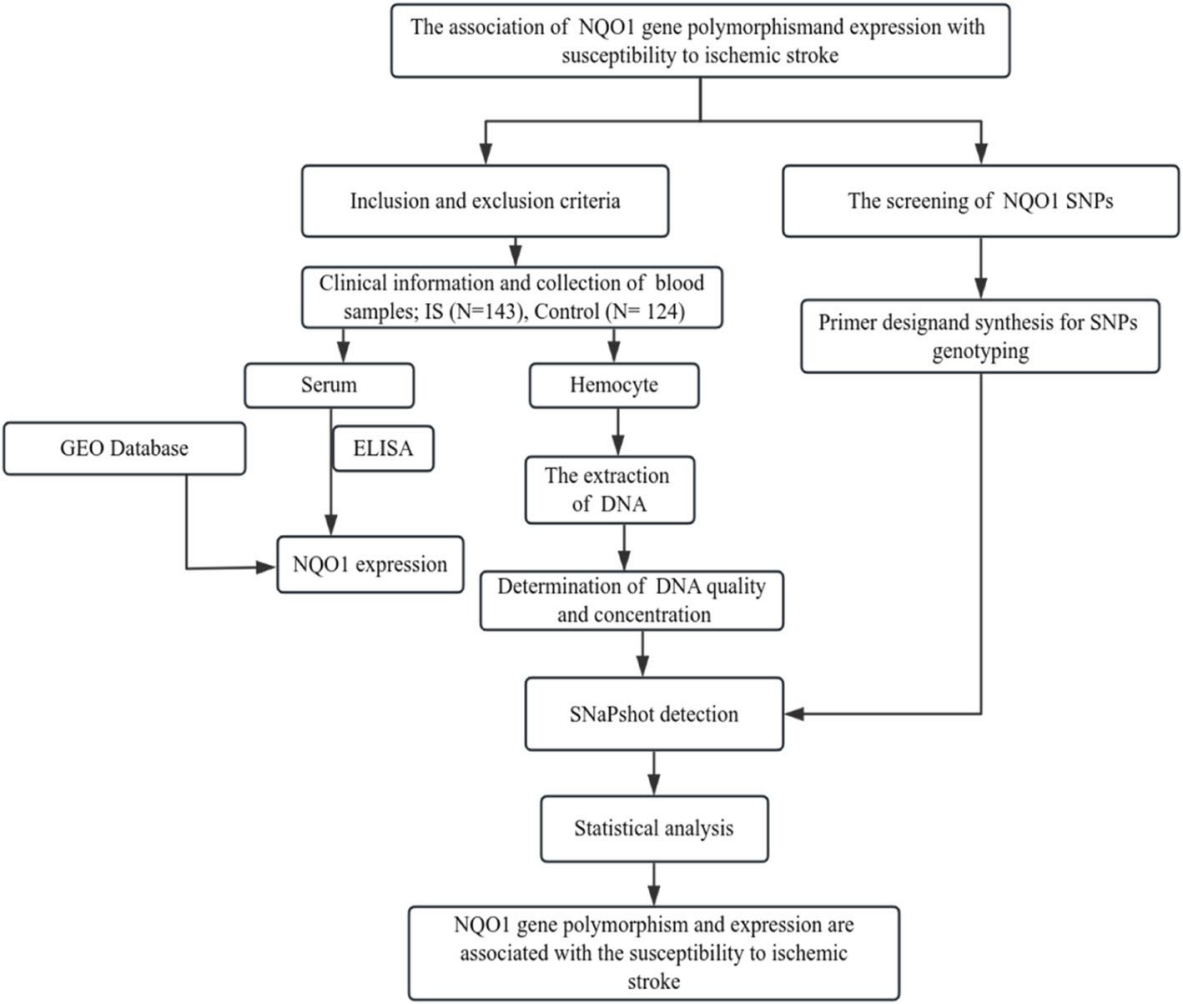
Workflow chart

Figure 1 shows the workflow of this study. The sample size was calculated based on the prediction model of PASS (2021, USA). The sample size was ≥ 74, and the actual study was 267 cases (control, *n* = 124; IS, *n* = 143). The study subjects were consecutively recruited from among individuals who underwent routine physical examinations at the Fourth People's Hospital of Yunnan Province from August 25, 2022, to October 28, 2022 (*n* = 124, 55.6% were male). Patients with IS hospitalized in the Department of Neurology were recruited consecutively (*n* = 143; 64.3% male). The inclusion criteria for the cerebral infarction group were patients who had lived in Yunnan for a long time, had been diagnosed with IS for the first time, and were ≥ 18 years old. The cerebral infarction group excluded patients with connective tissue disease, vasculitis, acute myocardial infarction, atrial fibrillation or heart valve disease, severe liver and kidney damage, severe infection, coagulation disorders, or recent use of drugs that affect coagulation function. The inclusion criteria for the physical examination group were routine physical examination personnel whose age and sex ratios matched those of the experimental group. The exclusion criteria for the physical examination team were those who had been diagnosed with IS or hemorrhagic stroke; those who suffered from neurological deficits, hereditary diseases, or severe cardiovascular and cerebrovascular diseases; and those who had recently used drugs that have adverse effects on coagulation function. All participants were required to provide data on age, sex, smoking history, drinking history, body mass index, 24 h ambulatory blood pressure, fasting blood sugar, blood lipid levels, platelet count, and past disease history. Data for the experimental group and their neurological examination scores during hospitalization were also collected. To collect peripheral venous blood from the participants, 2.0 mL of venous blood was drawn, mixed in an anticoagulant tube, and centrifuged at 1000 × g for 20 min. Plasma obtained after centrifugation was stored in enzyme-free centrifuge tubes, and all specimens were stored at − 80 °C.

### DNA extraction and genotyping

The SNP selection criteria were as follows: SNPs were those present in Homo sapiens, with a global minor allele frequency exceeding 5%, that had been published in the literature for analysis, and that had been published by the Thousand Genomes Project. According to the above criteria, three single nucleotide polymorphisms (rs2917673, rs689455, and rs1800566) of *NQO1* were screened from the NCBI dbSNP database (http://www.ncbi.nlm) and genotyped. A blood genomic DNA extraction kit (Tiangen Biochemical Technology) was used to extract genomic DNA. A spectrophotometer (IMPLEN, Munich, Germany) was used to measure the DNA concentration in the samples. These three SNPs were genotyped using SNaPshot on an ABI 3730XL sequencer (Applied Biosystems, 111 Waltham, MA, USA) [26–28]. The Illumina MiSeq high-throughput sequencing platform was used for high-throughput sequencing of the *NQO1* locus. All samples were stored at − 80 °C. Single nucleotide polymorphism (SNP) typing was used to analyze *NQO1* polymorphisms.

### Design and synthesis of substrates

Primers for the three SNPs in *NQO1* were designed using the Oligo software (version 7.37; https://www.oligo.net/), including polymerase chain reaction primers and unique bases. Extension primer design, primer synthesis, and genotype detection were carried out by the Anhui General Biotechnology Company in strict accordance with the experimental procedures (Tables 1 and 2).

**Table 1:** PCR amplification primer sequence

SNPs	Forward primer	Reverse primer
rs689455	5’-GGAGATATACTCTCAGTAGGTGAAG-3’	5’-CACTTTGTTGCTCAGGCTTTTC-3’
rs1800566	5’-GACTTACCTCTCTGTGCTTTC-3’	5’-AATACAGTGGTGTCTCATCCC-3’
rs2917673	5’-CCCTCGCCTAATCACCTATCA-3’	5’-ATGTTGCCCAGGCTGGTCAAATT-3’

*SNP* Single nucleotide polymorphism

**Table 2:** SNaPshot extension primer sequence

SNPs	Primer for extension	Extension direction
rs689455	TTTTTTTTTTTTTTTTTTTTTTTTTTTTTTTAACTGGTTTCCAACTCCCGACC	F
rs1800566	TTTTTTTTTTCTGTGGCTTCCAAGTCTTAGAA	F
rs2917673	GCTCACACCTATAATCCCAACA	F

*SNP* Single nucleotide polymorphism

### Expression analysis of NQO1 in public databases

The National Center for Biotechnology Information Gene Expression Omnibus (GEO) was searched using the keywords “ischemic stroke” and “*Homo sapiens*” (https://www.ncbi.nlm.nih.gov/geo/). After screening, GSE25414 (https://www.ncbi.nlm.nih.gov/gds/?term=GSE25414) and GSE37587 (https://www.ncbi.nlm.nih.gov/gds/?term=GSE37587) were used as target datasets, and the corresponding gene expression files were downloaded. The gene expression files were organized and merged to verify the expression of the target gene *NQO1*.

### Enzyme-linked immunosorbent assay (ELISA)

NQO1 levels in human peripheral blood were determined using a double-antibody one-step sandwich ELISA kit (Shanghai Keshun Biotechnology Co., Ltd., Shanghai, China). The OD was measured at a wavelength of 450.0 nm and converted to the solubility of the indicator to be measured.

### Statistical analysis

Statistical analyses were performed using SPSS version 22.0 (SPSS, Inc., Chicago, IL, USA) with α = 0.05. *P* < 0.05 indicated statistically significant differences between two datasets. *P* > 0.05 indicated that the two datasets complied with the law of genetic balance. The Hardy–Weinberg equilibrium (HWE) was used to analyze the population genetic balance of the samples. The sex, age, fasting blood glucose, blood lipid levels, and other measurement data were analyzed using the χ^2^ test; normality was also tested using the χ^2^ test. Student's t-test was used to analyze normally distributed data, and the Mann–Whitney U rank sum test was used to analyze non-normally distributed data. STRING and KEGG pathway analyses were performed to assess the biological activity and function of *NQO1*.

## Results

### Clinical characteristics of the study population

The clinical characteristics and results of the study population analysis are shown in Table 3. The average age of patients with IS (92 men and 51 women) was 63 years old; the average age of the control group (69 men and 55 women) was 60 years old. There were no statistically significant differences in age or sex between the IS and control groups (*P* > 0.05). White blood cells and fasting blood glucose in the IS group were higher than those in the control group (*P* < 0.05). The difference between the two groups was statistically significant. The high-density lipoprotein and apolipoprotein A1 levels in the IS group were lower than those in the control group (*P* < 0.05), and the difference between the two groups was statistically significant. Further analysis of clinical baseline data revealed that HDL was a protective factor for IS, with OR < 1. Blood glucose and apolipoprotein A1 levels were risk factors for IS (OR > 1) (Tables 3 and 4).

**Table 3:** Analysis of basic clinical characteristics of research subjects

Variables	Control (*n* = 124)	IS (*n* = 143)	*P*-value
Age (mean ± SD, year)	60[56.000,66.000]	63[53.000,71.000]	0.5520
Male/Female	69/55	92/51	
Height (m)	1.629 ± 0.081	1.643 ± 0.079	0.164
Weight (Kg)	64.771 ± 9.063	64.664 ± 11.949	0.934
WBC(10^9^/L)	5.916 ± 1.329***	7.519 ± 2.700	< 0.001
LDL-C (mean ± SD, mmol/L)	2.564 ± 0.734	2.435 ± 0.859	0.188
Triglycerides (mean ± SD, mmol/L)	2.132 ± 2.734	1.859 ± 1.531	0.274
HDL-C (mean ± SD, mmol/L)	1.429 ± 0.412***	1.174 ± 0.866	< 0.001
FBG (mean ± SD, mmol/L)	5.738 ± 1.664*	6.428 ± 3.572	0.002
Blood platelet count (10^9^/L)	218.50 ± 66.228	215.76 ± 76.373	0.754
Apolipoprotein A1 (mean ± SD, g/L)	1.623 ± 0.233*	1.064 ± 0.808	0.006
Apolipoprotein B (mean ± SD, g/L)	0.800 ± 0.190	0.793 ± 0.270	0.804

(*P < 0.05, **P < 0.01, ***P < 0.001)

*IS* Ischemic stroke, *WBC* White blood cell, *LDL-C* Low-density lipoprotein cholesterol, *HDL-C* High-density lipoprotein cholesterol, *FBG* Fasting blood glucose, *SD* Standard deviation

**Table 4:** Regression analysis of IS risk factors

Risk factors	*P*	OR	95%CI
WBC	< 0.001	1.602	1.315–1.953
HDL-C	0.212	0.436	0.118–1.607
Fasting blood glucose	0.068	1.150	0.990–1.336
Apolipoprotein A1	0.504	1.683	0.366–7.731
Constant	0.544	0.550	

*IS* ischemic stroke, *WBC* White blood cell,* P* *P* values, *OR* odds ratio, 95% *CI* 95% confidence interval, high-density lipoprotein cholesterol

### *NQO1* genetic polymorphisms

**Fig. 2:**
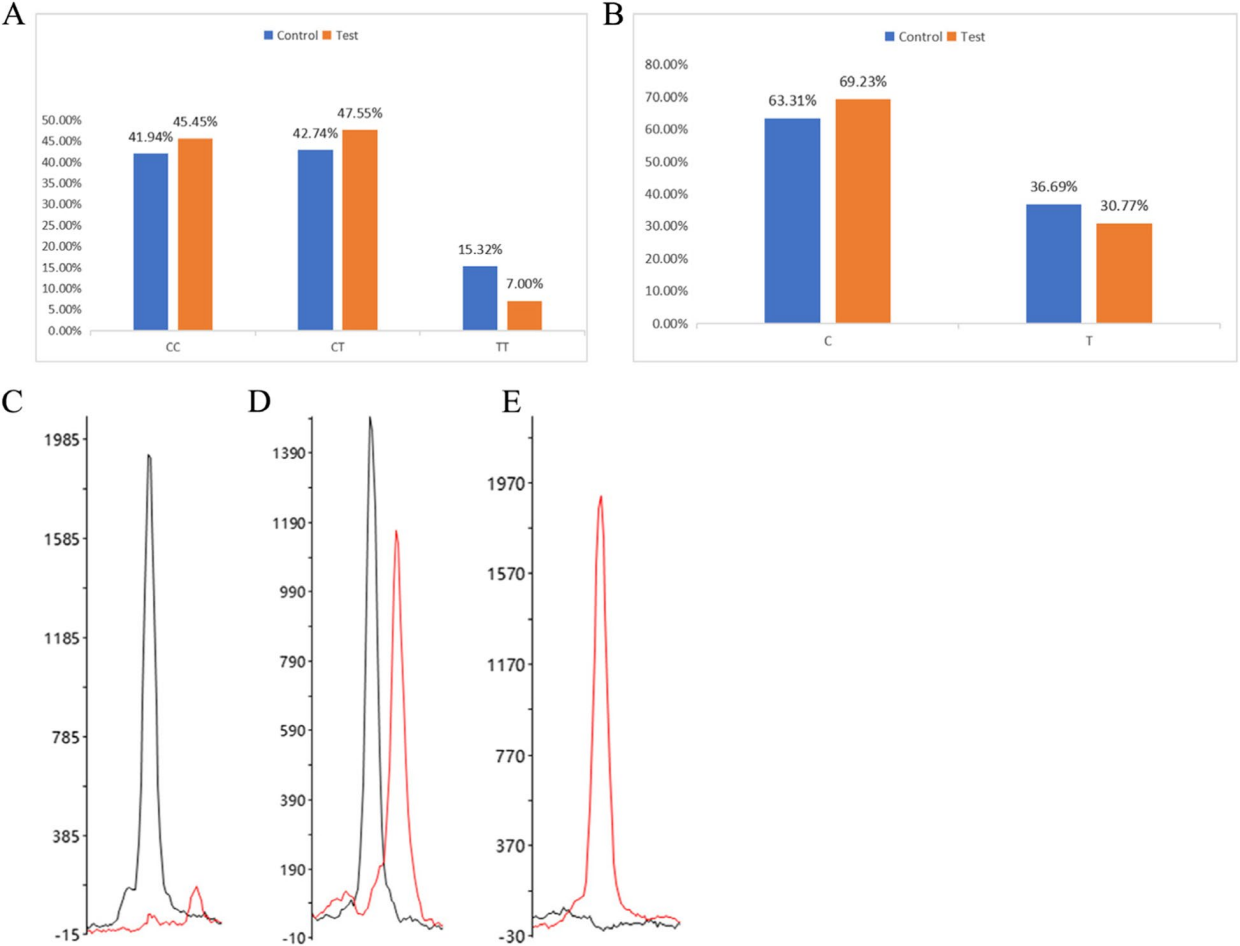
Histogram and representative peak diagram of genotyping of rs2917673 locus. **A** Genotypes of the rs2917673 locus in the control group and experimental group. **B** Histogram of allele frequency distribution of rs2917673 locus in control group and experimental group. **C** Representative CC genotype at rs2917673 locus. **D** Representative CT genotype of rs2917673 locus. **E** Representative TT genotype at the rs2917673 locus

The three SNP sites of *NQO1* in the Yunnan population were tested using the SNaPshot genotyping technology. The test results showed that rs2917673, among the three SNP sites of *NQO1*, harbors a genetic polymorphism and complies with the genetic balance test (Table 5). The rs2917673 locus had three genotypes: CC, CT, and TT. The genotype and allele frequency distribution histograms, genotyping, and peak diagrams of the rs2917673 locus in the control and experimental groups are shown in Fig. 2.

**Table 5:** HWE inspection

SNPs	Physical examination group			The IS group		
	N	χ^2^	HWE *P*-value	N	χ^2^	HWE *P*-value
rs689455						
TT	51			33		
GT	45	7.647	0.006	83	3.794	0.051
GG	28			27		
rs1800566						
CC	51			34		
CT	42	11.496	0	80	2.068	0.150
TT	31			29		
rs2917673						
CC	52			65		
CT	53	0.794	0.373	68	1.930	0.165
TT	19			10		

*HWE* Hardy–Weinberg equilibrium, *SNP* single nucleotide polymorphism, *IS* Ischemic stroke

### *NQO1* rs2917673 polymorphism and susceptibility to IS

Based on binary logistic regression analysis, the correlation between *NQO1* polymorphism sites and IS risk was analyzed. By constructing multiple genetic models, we analyzed whether they have an impact on the risk of IS. Genetic models included codominant, dominant, recessive, and super dominant models. The results of the analyses are presented in Table 6. The analysis indicated that TT carriers of the rs2917673 locus under the co-dominance model had an increased risk of IS compared with CC genotype carriers and were statistically associated with IS risk (*P* = 0.046). In the recessive gene model, TT gene carriers were associated with an increased risk of IS compared with CC + CT gene carriers (*P* = 0.033). Other genotypes of the rs2917673 locus were not associated with IS risk. Genotypes at the rs689455 and rs1800566 loci were excluded from the study because they did not meet the HWE criteria.

**Table 6:** Association analysis between gene locus polymorphisms and the risk of IS

SNPs	Model	Genetic typing	Physical examination group	The IS group	OR (95% CI)	*P*
	Codominant model	TT	51	33	Ref	
		GT	45	83	0.351[0.199–0.620]	< 0.001
		GG	28	27	0.671[0.338–1.333]	0.255
	The dominant model	TT	51	33	Ref	
		GT + GG	73	110	0.429[0.253–0.728]	0.002
rs689455	Recessive model	TT + GT	96	116	Ref	
		GG	28	27	1.253[0.692–2.269]	0.456
	Overdominant model	GG + TT	79	60	Ref	
		GT	45	83	0.412[0.251–0.675]	< 0.001
	allele model	T	147	149	Ref	
		G	101	137	0.747[0.530–1.054]	0.096
	Codominant model	CC	51	34	Ref	
		CT	42	80	0.350[0.197–0.620]	< 0.01
	The dominant model	TT	31	29	0.713[0.366–1.388]	0.319
		CC	51	34	Ref	
		CT + TT	73	109	0.446[0.264–0.755]	0.003
rs1800566	Recessive model	CC + CT	93	114	Ref	
		TT	31	29	1.310[0.737–2.330]	0.357
	Overdominant model	TT + CC	82	63	Ref	
		CT	42	80	0.403[0.245–0.663]	< 0.001
	allele model	C	144	148	Ref	
		T	104	138	0.775[0.550–1.091]	0.144
	Codominant model	CC	52	65	Ref	
		CT	53	68	0.974[0.584–1.625]	0.920
		TT	19	10	2.375[1.017–5.546]	0.046
	The dominant model	CC	52	65	Ref	
		CT + TT	72	78	1.154[0.710–1.875]	0.563
rs2917673	Recessive model	CC + CT	105	133	Ref	
		TT	19	10	2.407[1.073–5.396]	0.033
	Overdominant model	TT + CC	71	75	Ref	
		CT	53	68	0.823[0.507–1.336]	0.431
	allele model	C	157	198	Ref	
		T	91	88	1.304[0.910–1.870]	0.148

*SNP* Single nucleotide polymorphism, *IS* Ischemic stroke, *OR* Odds ratio, *95% CI* 95% confidence interval

### Expression analysis of *NQO1*

**Fig. 3:**
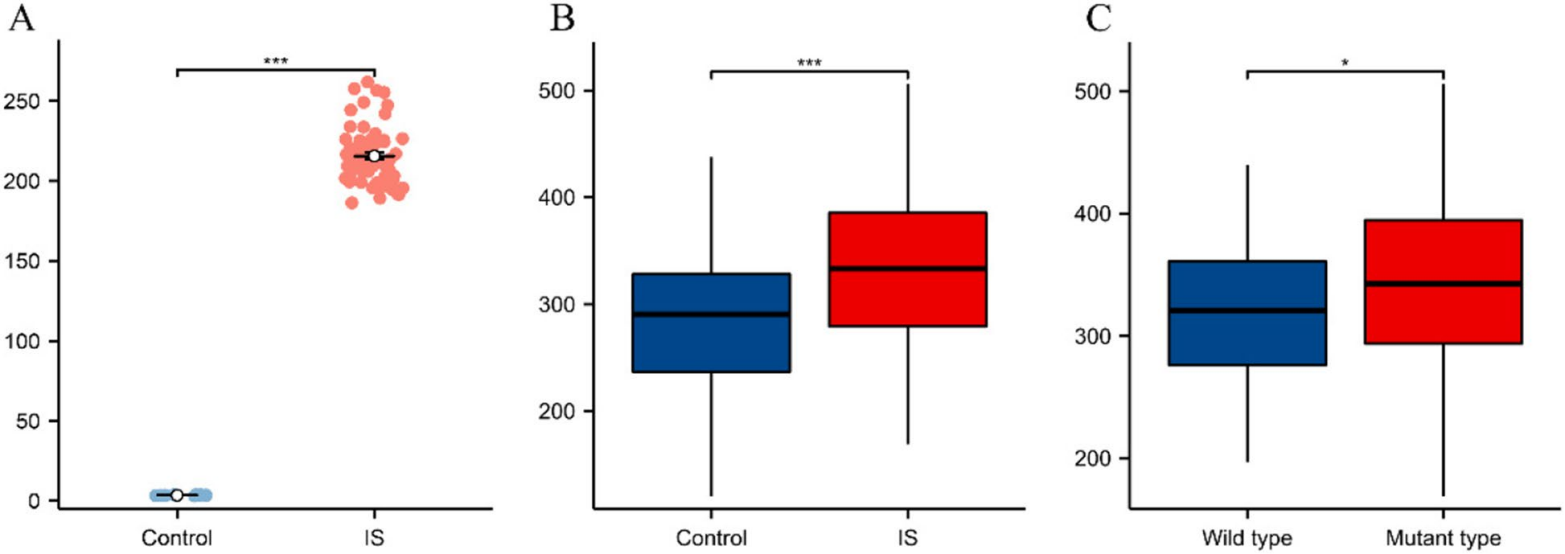
NQO1 expression in the Gene Expression Comprehensive Database and in peripheral blood. **A** Group differential expression in cerebral infarction data sets GSE25414 and GSE37587. *P* < 0.001. **B** Differences in NQO1 in peripheral blood between IS group and control group.^***^P < 0.001. **C** Differences in expression between wild type and mutant types of NQO1 rs2917673 locus in IS. ^*^P < 0.05

Analyses of public databases showed that the *NQO1* expression was upregulated in the GSE25414 and GSE37587 datasets (Fig. 3A). To further determine whether the polymorphisms in the rs2917673 locus affect the *NQO1* expression in patients with IS, ELISA was performed on patients' peripheral blood cells. The results showed differences in the expression of NQO1 between the IS and control groups with different genotypes at this site (*P* < 0.05, indicating a statistical difference), which was consistent with the bioinformatics analysis results (Fig. 3B). Intragroup comparison of the peripheral blood of IS patients revealed that between the wild-type (CC genotype) and mutant (CT/TT genotype) groups, the expression in the mutant group was slightly higher, and the difference between the two groups was statistically significant (*P* < 0.05) (Fig. 3C).

### *NQO1*-related functions

**Fig. 4:**
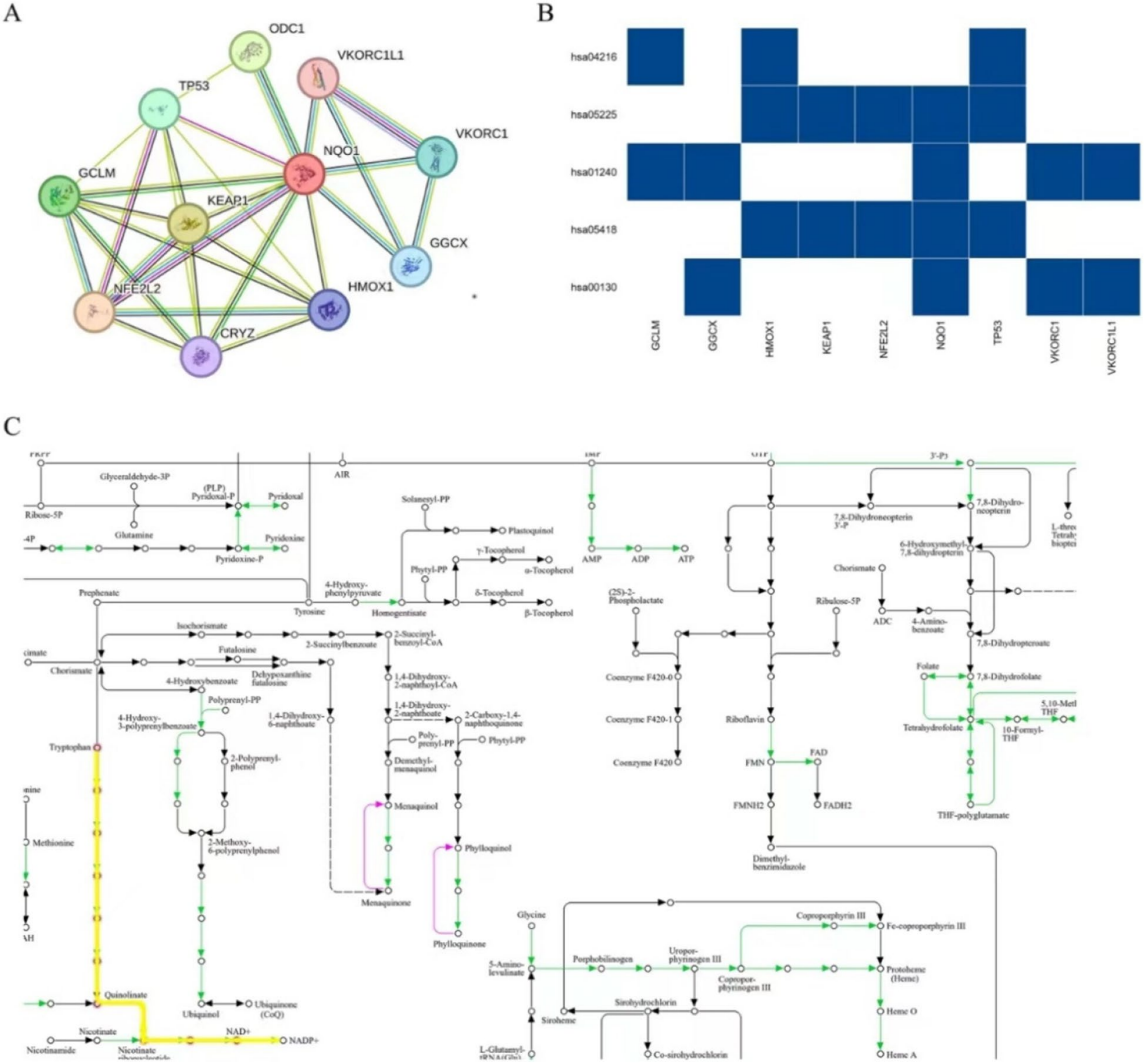
Biological functions of NQO1. **A** Protein interaction map of NQO1. **B** KEGG enrichment results of proteins related to NQO1. **C** The NADH or NADPH redox reaction signaling pathway in which NQO1 participates

To further explore the potential function of NQO1 in regulating IS, a protein–protein interaction map was constructed, and KEGG enrichment analysis was performed. Pathway enrichment analysis showed that NQO1 and its related proteins were mainly involved in ubiquinone and other terpenoid–quinone biosynthesis, fluid shear stress, atherosclerosis, and biosynthesis of cofactors, which are all closely related to IS (Fig. 4).

## Discussion

We found that white blood cell count, fasting blood glucose, and apolipoprotein A1 were risk factors for IS (OR > 1). High-density lipoprotein and apolipoprotein were protective factors against IS (OR < 1). The three polymorphic sites rs689455, rs1800566, and rs2917673 of *NQO1* in patients with IS and the control groups in Yunnan, China, were explored using genotyping technology. The rs689455 and rs1800566 polymorphic sites were not consistent with the HWE test and were not included in the study [29]. Carrying the TT genotype in codominant and recessive models of the rs2917673 locus is a risk factor for IS. Further research found that the expression level of *NQO1* in the peripheral blood was significantly higher in the IS group than in the control group. These results are consistent with the analysis of public databases. Intragroup comparisons of IS peripheral blood revealed that CT/TT mutations conferred susceptibility to IS. The study of genetic polymorphisms is conducive to individualized research on IS and contributes to IS prevention.

The brain consumes high levels of oxygen and is rich in polyunsaturated fatty acids, making it particularly susceptible to oxidative stress [30]. When IS occurs, the insufficient supply of oxygen and glucose in the cells of the brain leads to mitochondrial damage and excessive production of ROS as well as a post-ischemic neuroinflammatory response, which aggravates the progression of the ischemic area [31, 32]. The levels of antioxidant enzymes decrease when oxidative stress occurs in the brain. NQO1 is an antioxidant enzyme that plays an important role in oxidative stress in the brain [33]. NQO1 prevents quinones from participating in the quinone-catalyzed one-electron reduction reaction. Cytochrome P450 reductase protects cells from oxidative damage by generating semiquinone radicals and reactive oxygen species through electron reduction reactions [34, 35]. Multiple studies have reported the antioxidant, anti-inflammatory, and cytoprotective properties of NQO1 under neurological conditions. The NQO1 activator β-lapachone has anti-inflammatory effects under lipopolysaccharide (LPS)-induced neuroinflammatory conditions. Upregulation of *NQO1* expression also prevents hydrogen peroxide-induced apoptosis in primary cortical neurons [30].

Our study showed that the mutant type (CT/TT genotype) may be a risk factor for IS. SNPs in *NQO1* have profound phenotypic consequences [36, 37]. The lack of enzyme activity in cells homozygous for the proline-to-serine mutation in NQO1 and the C-to-T point mutation at position 609 was due to the absence of the NQO1 protein [37]. The NQO1 rs1800566 polymorphism is associated with coronary heart disease and atherosclerosis [38]. Atherosclerosis is the underlying cause of stroke, and HDL, apoA-I, and endogenous apoE prevent inflammation and oxidative stress and promote cholesterol efflux to reduce atherogenesis [39]. Atherosclerosis also promotes IS [32]. The genetic pathways related to NQO1 were mainly enriched in ubiquinone and other terpenoid–quinone biosynthesis pathways, fluid shear stress, atherosclerosis, and the biosynthesis of cofactors. Water-soluble quinone oxidoreductases are widely distributed in various human functions [40]. This process is closely related to the function of NQO1.

However, the present study had a few limitations. The samples of our study was limited to one hospital, and only three *NQO1* sites were studied. Future screening of multiple hospitals using epidemiological systematic sampling methods is needed to confirm this study. Further supports the relationship between polymorphisms of this gene and IS in this region. This may help prevent IS and develop individualized treatment strategies.

## Conclusions

The present study investigated three *NQO1* loci and found that the CT/TT genotype of rs2917673 is a risk factor for IS in Yunnan, China; *NQO1* rs2917673 polymorphism was found to be markedly associated with IS. Our findings will be of great value for the prevention and personalized treatment of IS in this region.

### Supplementary Information

Supplementary Material 1.

## Data Availability

The dataset used in this study is available from the Gene Expression Omnibus (GEO) Database (https://www.ncbi.nlm.nih.gov/geo/) was obtained. Database (https://www.ncbi.nlm.nih.gov/geo/) was obtained.

## Abbreviations

IS: Ischemic stroke
OR: Odds ratios
CI: Confidence interval
GEO: Gene Expression Omnibus
ELISA: Enzyme-linked immunosorbent assay
HWE: Hardy–Weinberg equilibrium
